# Efficient expansion of XCR1^+^CD141^+^ cDC1 cells from human PBMCs: a promising strategy for DC-based vaccine

**DOI:** 10.3389/fimmu.2026.1912573

**Published:** 2026-10-19

**Authors:** Ying Li, Kaiyun Huang, Rui Shang, Harunori Oda, Xinxin Gao, Xin Li, Yizhen Deng, Xiaodan Wang, Meng Ding, Can Song, Bin Wen, Xia Ling, Ming Shi, Hongjin Wu

**Affiliations:** 1 International Research Center for Regenerative Medicine, Boao International Hospital, Qionghai, Hainan, China; 2 School of Life Science and Technology, Harbin Institute of Technology, Harbin, Heilongjiang, China; 3 School of Life and Health Sciences, Hainan Province Key Laboratory of One Health, Collaborative Innovation Center of Life and Health, Hainan University, Haikou, Hainan, China; 4 Medical Corporation ISHIN-KAI ODA Clinic, Shinjuku-ku, Tokyo, Japan

**Keywords:** antigen-specific cytotoxic T lymphocytes (CTLs), conventional type 1 dendritic cells (cDC1s), IFN-γ, IL-12 cytokines, peripheral blood mononuclear cells (PBMCs), XCR1^+^ dendritic cells

## Abstract

**Introduction:**

Dendritic cells (DCs) are pivotal orchestrators of immune activation, serving as professional antigen-presenting cells that bridge innate and adaptive immunity. Among them, conventional type 1 dendritic cells (cDC1s) play a critical role in initiating cytotoxic T-cell responses and anti-tumor immunity. Despite their therapeutic promise, the clinical translation of cDC1-based vaccines has been limited by challenges in large-scale *in vitro* generation.

**Methods and results:**

Here, we established an efficient and reproducible two-step protocol for producing functional XCR1⁺ CD141⁺ cDC1s from human peripheral blood mononuclear cells (PBMCs), providing a practical and potentially scalable approach for cell sourcing. Transcriptomic profiling confirmed strong expression of cDC1 lineage markers and revealed that mature cDC1s exhibit pronounced activation of immune-related pathways and secretion of key cytokines and chemokines involved in immune modulation. Functionally, the generated cDC1s effectively primed antigen-specific cytotoxic T lymphocytes (CTLs), which exhibited significantly enhanced IFN-γ production and tumor cell cytotoxicity compared with both control T cells and monocyte-derived DC–induced CTLs (moDC-CTLs). Importantly, preclinical evaluation in NOG mice showed no treatment-related toxicity, providing preliminary—though model-limited—safety evidence for this approach.

**Discussion:**

Collectively, these findings establish an efficient and reproducible strategy for generating human cDC1s and highlight their potential as a candidate platform for dendritic cell–based cancer immunotherapy.

## Introduction

Dendritic cells (DCs) are pivotal regulators of immune activation and tolerance, functioning as professional antigen-presenting cells (APCs) that capture, process, and present antigens from pathogens and tumor cells to T lymphocytes (1). Through their ability to coordinate both innate and adaptive immune responses, DCs play a central role in antitumor immunity (2). Among the diverse DC subsets, conventional type 1 dendritic cells (cDC1s), characterized by their expression of XCR1 and CD141, are uniquely potent in cross-presenting antigens to CD8+ T cells, thereby driving robust cytotoxic T lymphocyte (CTL) responses crucial for antiviral and antitumor immunity. These intrinsic properties position cDC1s as highly attractive candidates for next-generation DC-based therapeutic vaccine development against cancer and infectious diseases, a strategy that has been extensively explored in clinical trials (3).

The accessibility of peripheral blood monocytes and well-established differentiation protocols have positioned monocyte-derived DCs (moDCs) as the most widely used subtype in DC-based vaccine studies. Most clinical DC vaccines rely on moDCs generated from peripheral blood monocytes using granulocyte-macrophage colony-stimulating factor (GM-CSF) and interleukin-4 (IL-4) (4, 5). Nonetheless, increasing evidence indicates that moDC-based vaccines induce suboptimal efficacy, partly due to their limited capacity for direct T-cell priming *in vivo (*6). In contrast, conventional type 1 DCs (cDC1s) represent a specialized subset with superior antigen cross-presentation and a distinct ability to prime cytotoxic CD8^+^ T cells, resulting in stronger antitumor responses compared with moDCs (7). These properties highlight cDC1s as promising candidates for next-generation DC vaccines.

However, the clinical translation of cDC1-based immunotherapies has been significantly hampered by the scarcity of these cells *in vivo* and the formidable challenges associated with their large-scale, *ex vivo* generation from human peripheral blood mononuclear cells (PBMCs) while maintaining their functional integrity. Despite their therapeutic potential, the clinical application of cDC1-based vaccines has been severely restricted by phenotypic heterogeneity and inconsistent functional activity. Primary cDC1s constitute less than 0.5% of peripheral blood mononuclear cells (PBMCs), making direct isolation impractical for clinical applications and representing a critical technical bottleneck in large-scale generation (8, 9). Existing methods to produce cDC1-like cells from hematopoietic stem cells or monocytes often require complex cytokine combinations and prolonged culture durations, suffer from complexity, low yield, and variability, limiting their clinical scalability and cost-effectiveness (10, 11).

In this study, we directly address this critical bottleneck by developing a simplified, efficient, and scalable two-step protocol for generating functional cDC1s from human PBMCs. This approach provides a feasible and scalable method that promotes the differentiation and expansion of XCR1 ^+^CD141^+^ cells with characteristic cDC1 phenotypic and functional properties. Our findings offer a practical strategy to overcome current manufacturing bottlenecks in DC-based immunotherapy and support the advancement of next-generation cancer vaccines with enhanced efficacy.

## Methods and materials

### Cell culture

Liver cancer cell line Hep G2 and Huh-7 were obtained from National Collection of Authenticated Cell Cultures (NCAC, Shanghai). Hep G2 cells were maintained in Modified Eagle Medium (MEM) supplemented with 10% fetal bovine serum (FBS), 1% NEAA, 100 U/mL penicillin, and 100 μg/mL streptomycin. Huh-7 cells were maintained in Dulbecco’s Modified Eagle Medium (DMEM) supplemented with 10% fetal bovine serum (FBS), 100 U/mL penicillin, and 100 μg/mL streptomycin. Cultures were incubated at 37 °C in a humidified atmosphere containing 5% CO_2_. For routine passaging, cells were grown to 80-90% confluency and harvested using 0.25% trypsin-EDTA solution. All cell culture reagents were purchased from Gibco (ThermoFisher Scientific, Waltham, MA).

### Human ethics statement

This study was approved by the BOAO International Hospital Institutional Review Board (IRB Approval Number: [BIH-2021-001]). All participants provided written informed consent prior to participation. The study was conducted in accordance with the ethical principles outlined in the Declaration of Helsinki. Participant confidentiality and privacy were protected throughout the research process.

### cDC1 manufacture and maturation

cDC1 is manufactured from PBMCs in two steps: Step 1, cell expansion: PBMCs were extracted and inoculated into cell culture medium StemSpan™-XF (STEMCELL, #100-0073) on T75 plates with a variety of cytokines (GMP cytokines from Bio-techne), including FLT3L (50 ng/ml), SCF (20 ng/ml), TPO (20 ng/ml), IL3 (10 ng/ml), IL6 (10 ng/ml) and SR1 (1 μmol/mL) (Stem Cell, # 72342); The medium was changed after 2 hours to remove suspended cells. Subsequently, the medium was changed every 3 days. After 7–9 days expansion, the attached cells will reach ~ 90% confluence in T75 plate and then proceed to the next step. Step 2, cell differentiation: The medium was changed to a differentiation medium, ImunoCult™-ACF Dendritic Cell Medium (STEMCELL, #10986) with a variety of cytokines (GMP cytokines from Bio-techne), including FLT3L (50 ng/ml), GM-CSF (5 ng/ml), SCF (10 ng/ml), IL3 (5 ng/ml), TGF-β (10 ng/ml); The medium was then changed every two days; After 5 days differentiation, the adherent cells were differentiated into suspension cells, which were immature cDC1 cells. cDC1 is maturated in ImunoCult™-ACF Dendritic Cell Medium with stimulation reagent mix [20 μg/ml Poly I:C (Sigma, I3036), 10 ng/ml R848 (Stem Cell, 4536/50), 5 ng/ml IFN-γ (R&D system, 285-GMP), 2.5 ng/ml TNF-α (R&D system, 210-GMP)]. Immature and mature cDC1 cells were further validated with flow cytometry.

### Donors and PBMC isolation

Peripheral blood was obtained from healthy adult donors at BOAO International Hospital. PBMCs were isolated by density-gradient centrifugation over Ficoll (TBD, #HY2015) according to the manufacturer’s instructions, washed twice with PBS, and counted before use. On average, approximately 1 × 10^6^ PBMCs were recovered per mL of whole blood. PBMCs used for phenotypic characterization were derived from 19 independent donors; functional CTL induction and cytotoxicity experiments were performed with cells from two donors (Sample #1, 65 years; Sample #2, 25 years).

### Monocyte-derived DC generation

Monocyte-derived dendritic cells (moDCs) were generated from the adherent, monocyte-enriched fraction of PBMCs cultured in the presence of GM-CSF and IL-4, and subsequently matured according to established protocols (4, 5). Phenotypic validation of immature and mature moDCs was performed by flow cytometry before proceeding with T-cell stimulation experiments.

### Flow cytometry and antibodies

For immunophenotypic analysis, cells were harvested and washed twice with phosphate-buffered saline (PBS) containing 2% fetal bovine serum (FBS). Cells were stained with the following directly conjugated monoclonal antibodies: CD3-FITC/CD16+CD56-PE/CD45-PerCP (#340300), CD4-FITC/CD8-PE/CD3-PerCP (#340298), anti-CD56 (#556647), anti-CD16 (#561308), anti-CD279 (#558694), anti-CD141 (#564123), anti-CD14 (#555397) and IgG1, κ Isotype Control antibodies from BD Biosciences (San Jose, CA, USA); anti-XCR1 (R&D Systems, FAB8571P-100). Samples were stained and data were acquired according to the manufacturer’s instructions (BD Accuri™ C6 Plus flow cytometer). Data were analyzed using FlowJo software (version 10.8.1, Tree Star Inc.).

### RNA sequencing

Total RNA was isolated from cells using the RNeasy Mini Kit (Qiagen, #74104) according to the manufacturer’s instructions. RNA quality and integrity were assessed using an Agilent 2100 Bioanalyzer with RNA 6000 Nano chips (Agilent Technologies), and only samples with RNA Integrity Number (RIN) > 7.0 were used for library preparation. Libraries were constructed using the NEBNext Ultra II Directional RNA Library Prep Kit for Illumina (New England Biolabs, #E7760L) with poly(A) mRNA selection. Libraries were sequenced on an Illumina NovaSeq 6000 platform with 150 bp paired-end reads, generating an average of 20 million reads per sample. Raw sequencing data (FASTQ files) were processed using the following bioinformatics pipeline: quality control with FastQC v0.11.9, adapter trimming with Trimmomatic v0.39, and alignment to the human reference genome (GRCh38/hg38) using STAR v2.7.9a with default parameters. Gene-level read counts were generated using featureCounts v2.0.1 with GENCODE v39 annotation. Differential gene expression analysis was performed using DESeq2 v1.34.0 in R v4.1.2, with genes considered significantly differentially expressed at an adjusted p-value < 0.05 and absolute log2 fold change > 1.0 (12). Functional enrichment analysis was conducted using clusterProfiler v4.2.2 with Gene Ontology (GO) and Kyoto Encyclopedia of Genes and Genomes (KEGG) databases (13). RNA-seq was performed on cells derived from independent donors (three biological replicates per condition), and all libraries were prepared and sequenced in a single batch to minimize batch effects; differential-expression comparisons between immature and mature cDC1s used a donor-matched (paired) design with donor as a blocking factor.

### T cell receptor sequencing

T cells were collected and total RNA was extracted using the RNeasy Mini Kit (Qiagen, #74104) according to the manufacturer’s instructions. TCR α and β chain libraries were constructed using a 5’ RACE-based approach targeting the variable (V) and constant (C) regions of the TCR genes. Libraries were purified using AMPure XP beads (Beckman Coulter, #A63881), and size selection was performed to enrich for TCR CDR3-containing fragments. Libraries were sequenced on an Illumina NovaSeq 6000 platform with 150 bp paired-end reads, generating an average of 5 million reads per sample. Raw sequencing data were processed using the MiXCR v3.0.13 software pipeline with default parameters. Sequences were filtered for quality (Phred score > 20), UMIs were deduplicated, and TCR clonotypes were defined based on unique combinations of V, D, J gene segments and CDR3 nucleotide sequences. Productive rearrangements were identified by the presence of in-frame CDR3 sequences. Clonotype frequencies were calculated as the proportion of each unique clonotype relative to the total number of productive rearrangements. TCR repertoire diversity was analyzed using the Chao1 estimator and Shannon-Wiener index implemented in the Immunarch v0.6.5 R package (14).

### Enzyme-linked immunosorbent assay

ELISA was performed to quantify interleukin-12 (IL-12) and Interferon-γ (IFN-γ) levels in cell culture supernatants using the Human IL-12p70 ELISA Kit (ACRO biosystem, CRS-B009) and Human Interferon-γ (IFN-γ) ELISA Kit (ACRO biosystem, CRS-A017) according to the manufacturer’s instructions. Absorbance was measured at 450 nm with wavelength correction at 570 nm using an Infinite E Plex (TECAN, Switzerland). Standard curves were generated using four-parameter logistic regression (R² > 0.99), and IL-12 and IFN-γ concentrations were calculated by interpolating sample absorbance values from the standard curve. The lower limit of detection was 2.0 pg/mL for IL-12 and 5.309 pg/mL for IFN-γ, and intra-assay and inter-assay coefficients of variation were <10% and <15%, respectively.

### Enzyme linked immunospot assay

cDC1/moDC stimulated or no stimulated T cells (CTLs) were collected and washed twice with PBS, resuspended in complete medium, and adjusted to a concentration of 1×10^6^ cells/ml. IFN-γ ELISpot assays were performed to detect antigen-specific T cell responses using the Human Interferon-γ (IFN-γ) ELISPOT Kit (ACRO biosystem, RAS-SP001) according to the manufacturer’s instructions. Plates were air-dried and spots were counted using an Autoimmun Diagnostika GmbH (AID, Germany).

### Cytotoxicity of CTLs

The cytotoxicity of cDC1/moDC stimulated or no stimulated T cells (CTLs) were assessed by measuring the mean fluorescence intensity of Calcein AM (#C3099; Thermo Fisher Scientific, Waltham, MA, USA) in intact cancer cells using Terascan VPC (Minerva Tech, Tokyo, Japan). Briefly, target cells (liver cancer cell line HepG2 or Huh 7) were resuspended with Dulbecco’s modified Eagle’s medium containing 10% fetal bovine serum, and mixed with 20 μL of Calcein AM at 37 °C for 30 min for staining. CTLs were co-cultured with pre-stained target cells at a particular E:T ratio for 2 h or 4 h. The mean fluorescence intensity of living target cells was measured. The percent cytotoxicity of cells was calculated using the following formula: % cytotoxicity = (1 − [(average fluorescence of the sample wells − average fluorescence of the maximal-release control wells)/(average fluorescence of the minimal-release control wells − average fluorescence of the maximal-release control wells)]) ×100 (15).

### Animal study design

NOG mice were randomly allocated to four groups (n = 10 per group; 5 males and 5 females): Vehicle Control (PBS), Antigen Control (GPC3 antigen), cDC1-LD (1 × 10^6^ cells/kg), and cDC1-HD (1 × 10^7^ cells/kg). Cells were administered as a single subcutaneous injection. Animals were monitored for 14 days for body weight, food intake, clinical signs, hematology, plasma biochemistry, serum cytokines, biodistribution, and histopathology. Investigators were not blinded to group allocation.

### Ethics statement

All animal experiments were performed in accordance with the guidelines of the Hainan University Animal Use and Care Committee (HNUAUCC), and approved by the HNUAUCC (Approval Number: [HNUAUCC-2025-00374]). All procedures were carried out in compliance with the ARRIVE guidelines and the National Institutes of Health Guide for the Care and Use of Laboratory Animals.

### Statistical analyses

The experimental unit was defined as cells derived from an individual donor; technical replicates were not treated as independent biological replicates. The number of biological replicates (independent donors or independent cell preparations) and technical replicates for each experiment is reported in the corresponding figure legend. Data are presented as mean ± SD. Comparisons between two groups were performed using two-tailed unpaired Student’s t-tests, or paired t-tests for donor-matched samples. Comparisons among three or more groups were performed using one-way ANOVA followed by Tukey’s multiple-comparisons test, and time-course data were analyzed by two-way ANOVA followed by Šídák’s multiple-comparisons test. For RNA-seq, differential expression was assessed with the DESeq2 Wald test, and p-values were adjusted using the Benjamini–Hochberg procedure. Graphs were plotted and analyzed with Prism 7.1 (GraphPad, La Jolla, CA, USA). P < 0.05 was considered significant.

### Availability of data and material

All data have been added in the Supplementary Figures and Tables. The raw sequence data were uploaded to GEO with the accession number GSE313610.

## Results

### Establishment of a clinically scalable protocol for generating functional cDC1 cells from PBMCs

To establish a clinically viable platform for generating functional conventional type 1 dendritic cells (cDC1s; XCR1^+^CD141^+^) from human peripheral blood mononuclear cells (PBMCs), we optimized a cytokine-driven differentiation system. Multiple cytokine combinations, including FLT3L, GM-CSF, SCF, IL-3, IL-4, and TGF-β, were systematically evaluated for their ability to promote cDC1 differentiation. Among these, the formulation designated FGS3T [containing 50 ng/mL FLT3L, 5 ng/mL GM-CSF, 10 ng/mL SCF, 5 ng/mL IL-3, and 10 ng/mL TGF-β] consistently produced over 80% XCR1^+^CD11b^-^ cDC1s from PBMCs (**Supplementary Figure 1**). Despite high purity, this direct differentiation method showed considerable donor-to-donor variability and relatively low yields, limiting its scalability for clinical manufacturing.

To overcome these limitations, we further developed a two-stage manufacturing process incorporating a PBMC pre-expansion phase prior to differentiation (**Figures 1A, B**). Screening various cytokine formulations identified H5-S and L5-S (50 ng/mL FLT3L, 20 ng/mL SCF, 20 ng/mL TPO, 10 ng/mL IL-3, 10 ng/mL IL-6, and 1 µmol/L SR1) as optimal for expanding cDC1 precursors within PBMCs (**Supplementary Figures 2A, B**). When combined with the differentiation step, this protocol reproducibly yielded >70% XCR1^+^CD141^+^ cDC1s (**Figure 1C**; **Supplementary Figure 2C**). For maturation, a range of TLR agonists was tested. The combination of poly(I:C) and R848 induced the most robust phenotypic and functional maturation (**Supplementary Figures 3A, B**). Cytokine profiling identified IFN-γ as essential for terminal maturation, whereas TGF-β was dispensable (**Supplementary Figure 3C**). The inclusion of synthetic peptide antigens during maturation did not alter differentiation efficiency (**Supplementary Figure 4**).

**Figure 1:**
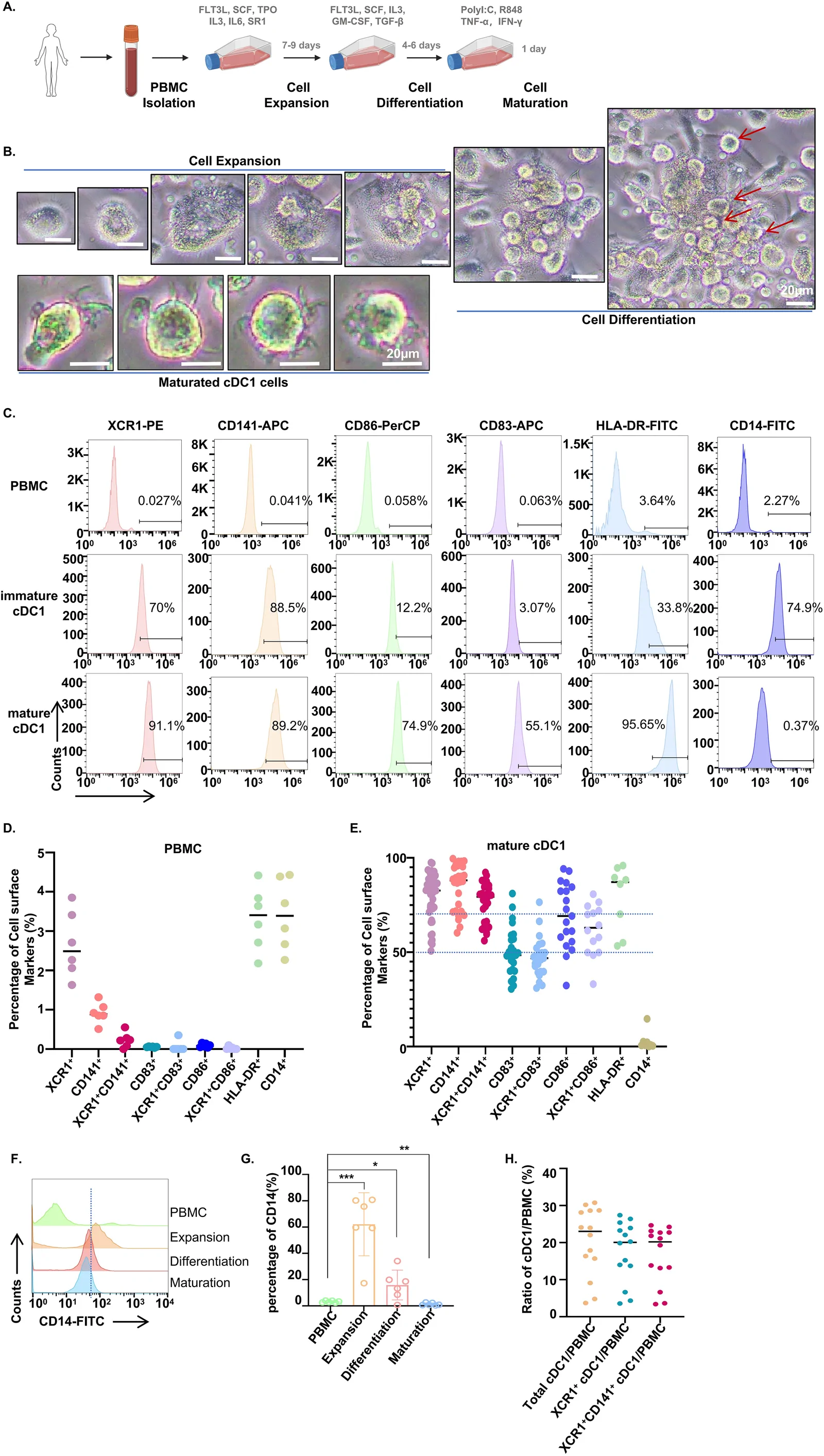
Novel two-stage manufacturing platform for cDC1 cells using PBMCs. **(A)** Diagram Flowchart of our two-step protocol for manufacturing cDC1 cells with PBMCs. Step 1, cell expansion [Cytokines: 50 ng/mL Flt3L, 20 ng/mL SCF, 20 ng/mL TPO, 10 ng/mL IL-3, 10 ng/mL IL-6, and 1 µmol/L SR1]; Step 2, cell differentiation [Cytokines: 50 ng/mL Flt3L, 5 ng/mL GM-CSF, 10 ng/mL SCF, 5 ng/mL IL-3, and 10 ng/mL TGF-β]; Maturation: poly I:C, 20 µg/ml, R848–10 ng/ml, IFN-γ 5 ng/ml, TNF-α 2.5 ng/ml, antigen peptides 20 µg/ml. **(B)** The morphological changes of cDC1 cells *in vitro* culture at different stages encompassing expansion, differentiation and maturation. Scale bar: 20 µm. **(C)** The proportion of surface markers (XCR1, CD141, CD86, CD83, HLA-DR and CD14) in PBMC, immature cDC1 and mature cDC1 was analyzed by flow cytometry. **(D, E)** Statistical analysis of cell surface markers in PBMC, n=6 **(D)** and mature cDC1, n>8 **(E)**. **(F)** Flow cytometric analysis of CD14 expression at different stages during *in vitro* cDC1 manufacturing. **(G)** Statistical analysis of CD14 expression at different stages during *in vitro* cDC1 manufacturing. *p < 0.05, **p < 0.01, ***p < 0.001. **(H)** The yield of cDC1 cells cultured *in vitro* (relative to initial PBMCs). Data are presented as mean ± SD; n represents independent donors. Statistical comparisons in **(G)** were performed by one-way ANOVA followed by Tukey’s multiple-comparisons test.

Phenotypic characterization of the resulting cDC1s revealed high expression of XCR1 and CD141 (~70%), CD83 and CD86 (~50%), and HLA-DR (≥90%), with minimal CD14 (<5%), confirming the absence of monocyte contamination (n = 19; **Figures 1D, E**). Longitudinal monitoring of CD14 expression showed transient upregulation during expansion followed by progressive downregulation during differentiation and maturation (**Figures 1F, G**), consistent with a monocytic origin.

The optimized two-step process achieved a conversion efficiency of ~20% of input PBMCs into mature cDC1s (**Figure 1H**). From only 60 mL of peripheral blood (~6 × 10^7^ PBMCs), approximately 1.2 × 10^7^ functional cDC1s were produced, corresponding to roughly 2 × 10^5^ cDC1s per mL of peripheral blood; in other words, approximately 50 mL of peripheral blood is sufficient to generate 1 × 10^7^ cDC1s, a yield within the range required for clinical vaccine dosing. This streamlined and reproducible protocol provides a robust platform for generating phenotypically stable and functionally active cDC1s suitable for next-generation DC-based cancer immunotherapies.

### Transcriptomic profiling reveals robust functional maturation and a distinct molecular identity of *in vitro*–generated cDC1 cells

To define the molecular characteristics of *in vitro*–generated cDC1s, we performed RNA sequencing on immature and mature cell populations. Transcriptomic analysis revealed extensive transcriptional remodeling during maturation, with 1,675 genes upregulated and 1,331 genes downregulated in mature compared with immature cDC1s (**Figure 2A**). Key maturation markers—including CD83, CD80, CD86, CCR7, CD40, and CCR5—were markedly upregulated (**Figure 2B**), indicating enhanced T-cell stimulatory and migratory capacities. Lineage-defining transcription factors BCL6 (16), IRF8 (17), BATF3 (18), KLF4 (19), as well as newly defined cDC1 functional marker MS4A7 (20) were stably expressed in both states (**Figure 2C**), confirming maintenance of cDC1 identity throughout differentiation. Mature cDC1s displayed elevated expression of immune-activating cytokines and chemokines (IL-1, IL-10, IFN-γ, CXCL9, CXCL10) (**Figures 2D, E**), as well as strong expression of HLA class I and II genes (**Figure 2F**), consistent with enhanced antigen presentation capability.

**Figure 2:**
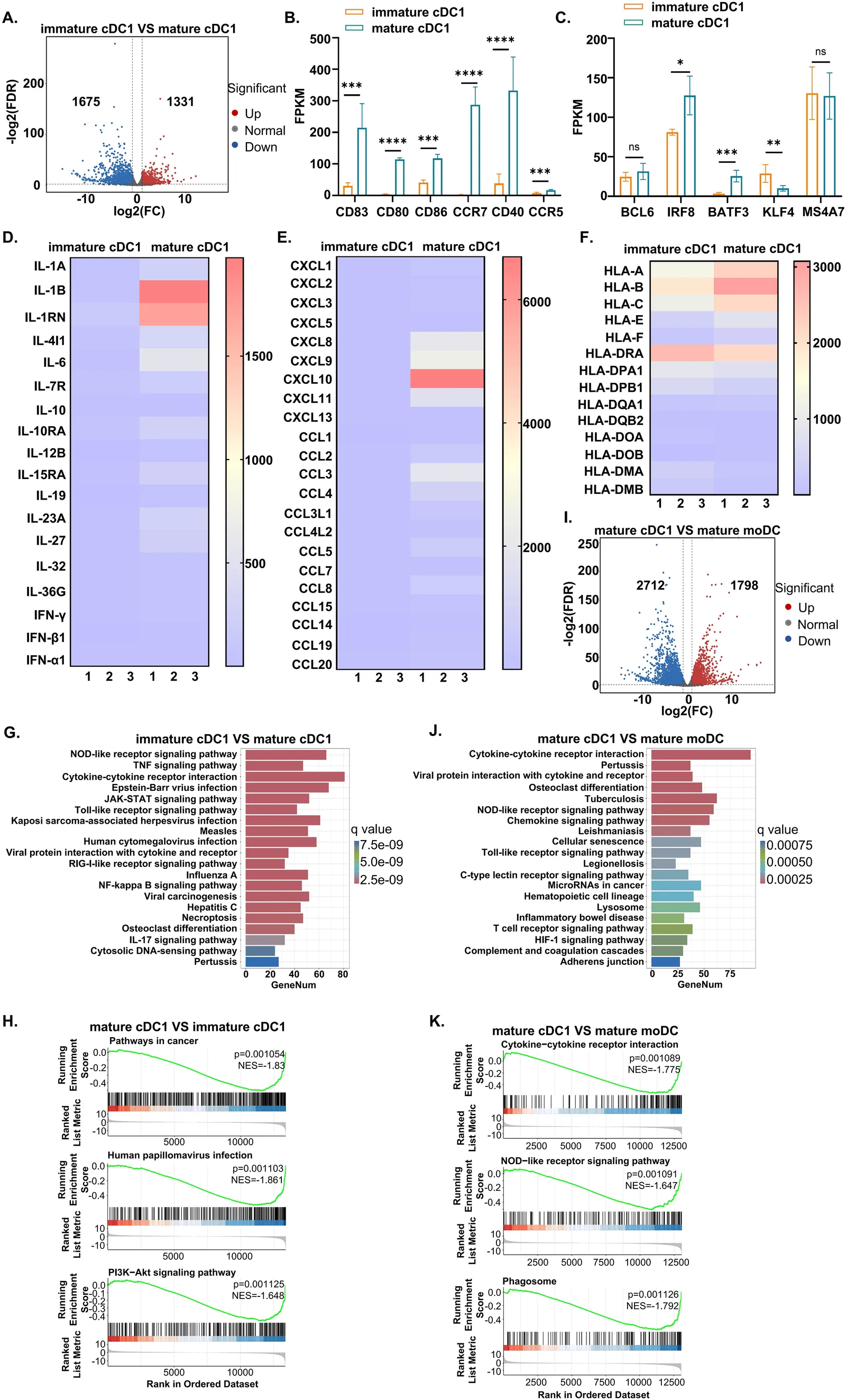
Advanced molecular characterization of *in vitro* cultured cDC1 cells using high-throughput RNA sequencing. **(A)** The volcano plot showed the difference of transcriptional expression between *in vitro* cultured immature cDC1 and mature cDC1. **(B, C)** The expression levels of dendritic cell markers differed between *in vitro* cultured immature and mature cDC1, including surface markers **(B)** and transcription factor markers **(C)**. ns, not significant, **p < 0.01, ***p < 0.001, ****p < 0.0001. **(D, E)** The heatmap reveals the expression differences of immune-related factors in *in vitro* cultured immature and mature cDC1, including cytokines **(D)** and chemokines **(E)**. **(F)** The heatmap showed the expression differences of HLA in *in vitro* cultured immature and mature cDC1. **(G)** KEGG pathway analysis of differentially expressed genes (DEGs) between *in vitro* cultured immature and mature cDC1. **(H)** Gene set enrichment analysis (GSEA) of DEGs between *in vitro* cultured immature and mature cDC1. **(I)** The volcano plot showed the difference of transcriptional expression between *in vitro* cultured mature cDC1 and mature moDC. **(J)** KEGG pathway analysis of *in vitro* cultured mature cDC1 and moDC. **(K)** GSEA of DEGs between *in vitro* cultured mature cDC1 and mature moDC. RNA-seq was performed on three biological replicates (cells from independent donors) per condition, sequenced in a single batch; differential expression was analyzed with DESeq2 using a donor-matched design (Benjamini–Hochberg adjusted p < 0.05).

Pathway enrichment analyses further confirmed functional activation. KEGG analysis identified significant enrichment of immune-related pathways, including NOD-like receptor signaling, cytokine–cytokine receptor interaction, and TNF signaling (**Figure 2G**). GSEA revealed activation of gene sets associated with cancer immunity, HPV infection, and PI3K–Akt signaling (**Figure 2H**), underscoring their immunotherapeutic potential.

Comparative transcriptomics between cDC1s and monocyte-derived DCs (moDCs) demonstrated pronounced molecular divergence, with 1,798 genes upregulated and 2,712 downregulated in moDCs relative to cDC1s (**Figure 2I**). KEGG and GSEA analyses showed preferential enrichment of immune pathways—including cytokine signaling, chemokine signaling, and phagosome formation—in cDC1s (**Figures 2J, K**). Collectively, these data confirm that our *in vitro* – manufactured cDC1s possess a unique molecular signature distinct from moDCs, defined by enhanced transcriptional programs for antigen processing, cytokine signaling, and immune activation, supporting their role as a specialized immunotherapeutic cell type.

### Generation of antigen-specific T cells by *in vitro*–manufactured cDC1 cells

To assess the antigen-presenting function of *in vitro*–generated cDC1s, we co-cultured GPC3 peptide (TIHDSIQYV)-loaded cDC1s (1 × 10^6^ cells) with monocyte-depleted PBMCs (1 × 10^7^) from two donors (Sample #1: 65 years; Sample #2: 25 years) to induce antigen-specific cytotoxic T lymphocytes (cDC1-CTLs) (**Figure 3A**). TCR repertoire sequencing revealed strong antigen-specific T-cell activation. TRBV/TRBJ usage analysis showed no activation in antigen-alone controls, whereas GPC3-loaded cDC1s triggered diverse antigen-reactive T-cell populations (**Figure 3B**). CDR3 tree mapping demonstrated distinct clonal expansion patterns in cDC1-CTL and cDC1-CTL+antigen (M) groups (**Figure 3C**).

**Figure 3:**
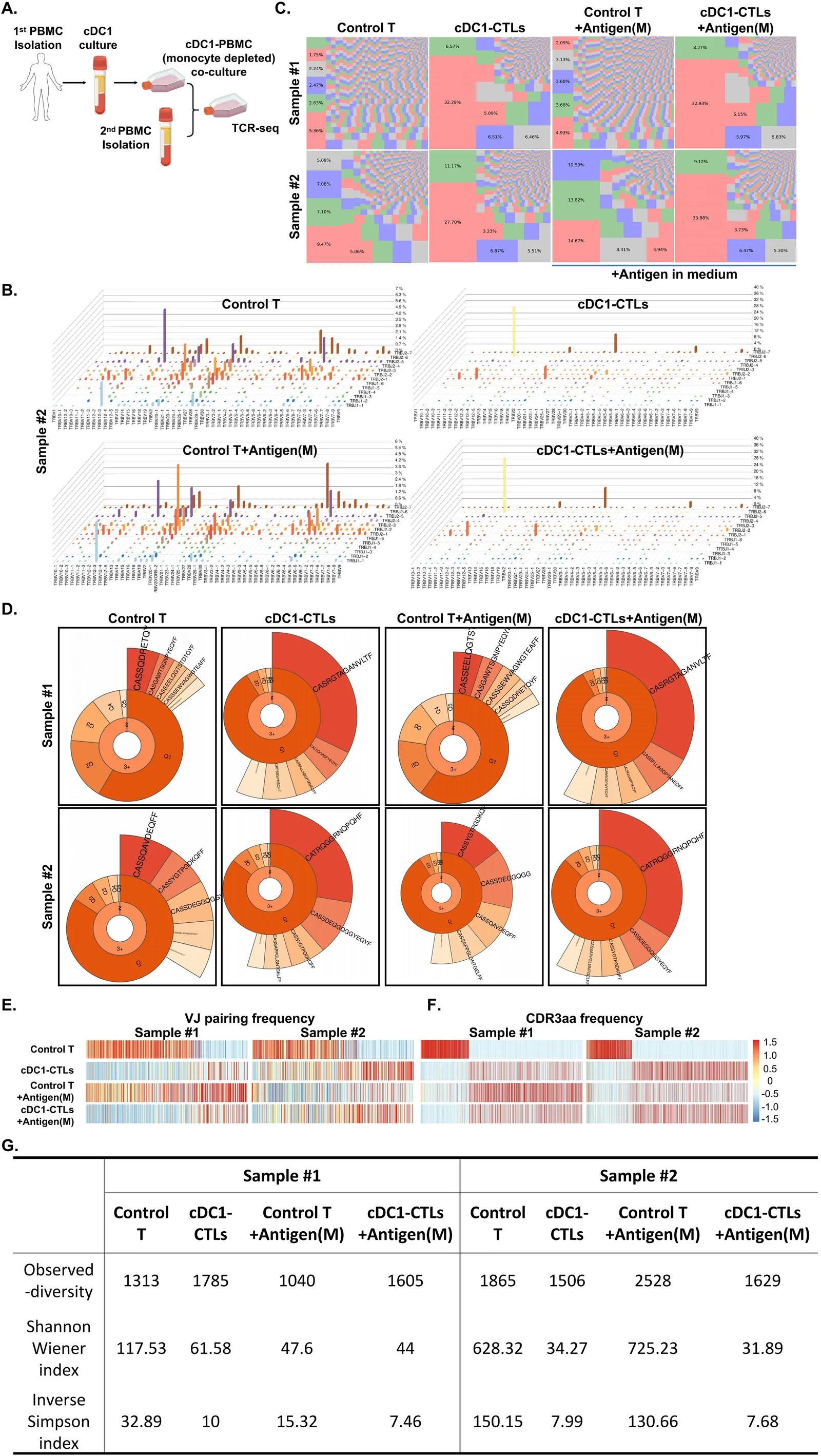
TCR sequencing analysis of cDC1-CTLs. **(A)** Schematic illustration of cDC1-CTLs production. **(B)** Heatmap depicting TRBV/TRBJ usage patterns from TCR sequencing data of cDC1-CTLs derived from two volunteers. Experimental groups: Control T, T cells cultured without cDC1 stimulation; cDC1-CTLs, T cells cultured with cDC1 stimulation; Control T+Antigen(M), T cells cultured without cDC1 stimulation but with antigens added to the culture medium; cDC1-CTLs+ Antigen(M), T cells cultured with cDC1 stimulation and antigens added to the culture medium. **(C)** Bar chart showing CDR3 tree mapping analysis of TCR sequencing data from cDC1-CTLs of volunteer #2. **(D)** Circular diagram representing the TCR repertoire diversity of cDC1-CTLs from two volunteers. **(E, F)** Heatmaps illustrating VJ pairings **(E)** and CDR3 amino acid frequency distributions **(F)** in cDC1-CTLs from both volunteers. VJ pairings and CDR3 amino acid frequency distributions showed significant specificity in cDC1-stimulated T cells, with partial specificity observed in antigen-stimulated T cells. **(G)** Diversity indices including observed diversity, Shannon-Wiener analysis, and Inverse Simpson indices of antigen-specific clones identified in the TCR sequencing data of the two volunteers. Data were generated from two donors (Sample #1 and Sample #2) and are displayed separately for each donor.

Diversity analyses revealed a marked contraction of the TCR repertoire following cDC1 stimulation, consistent with antigen-driven clonal expansion (**Figure 3D**). Heatmaps of VJ pairings and CDR3 amino acid frequencies confirmed enrichment of dominant clonotypes, particularly in Sample #2 (younger donor) (**Figures 3E,F**). Shannon–Wiener and Inverse Simpson indices both decreased significantly (**Figure 3G**), further supporting selective amplification of antigen-specific clones. Data from the two donors were analyzed and are displayed separately in all panels of **Figure 3**. Together, these results demonstrate that *in vitro*–generated cDC1s efficiently prime and expand antigen-specific CTLs, eliciting focused, high-affinity immune responses and highlighting their potential as potent cellular adjuvants for cancer vaccination.

### *In vitro*–generated cDC1 cells exhibit potent immunostimulatory activity and induce functional CTLs with robust antitumor effects

To evaluate the immunostimulatory potential of cDC1s, we quantified IL-12 secretion, a key indicator of dendritic cell activation. Immature cDC1s did not produce IL-12 (IL-12<20pg/mL), while mature cDC1s produced high IL-12 levels—approximately 9 ng/mL per 1×10^5^ cells (Sample #1) and 7 ng/mL per 1×10^5^ cells (Sample #2) (**Figure 4A**)— demonstrating strong intrinsic immunogenicity.

**Figure 4:**
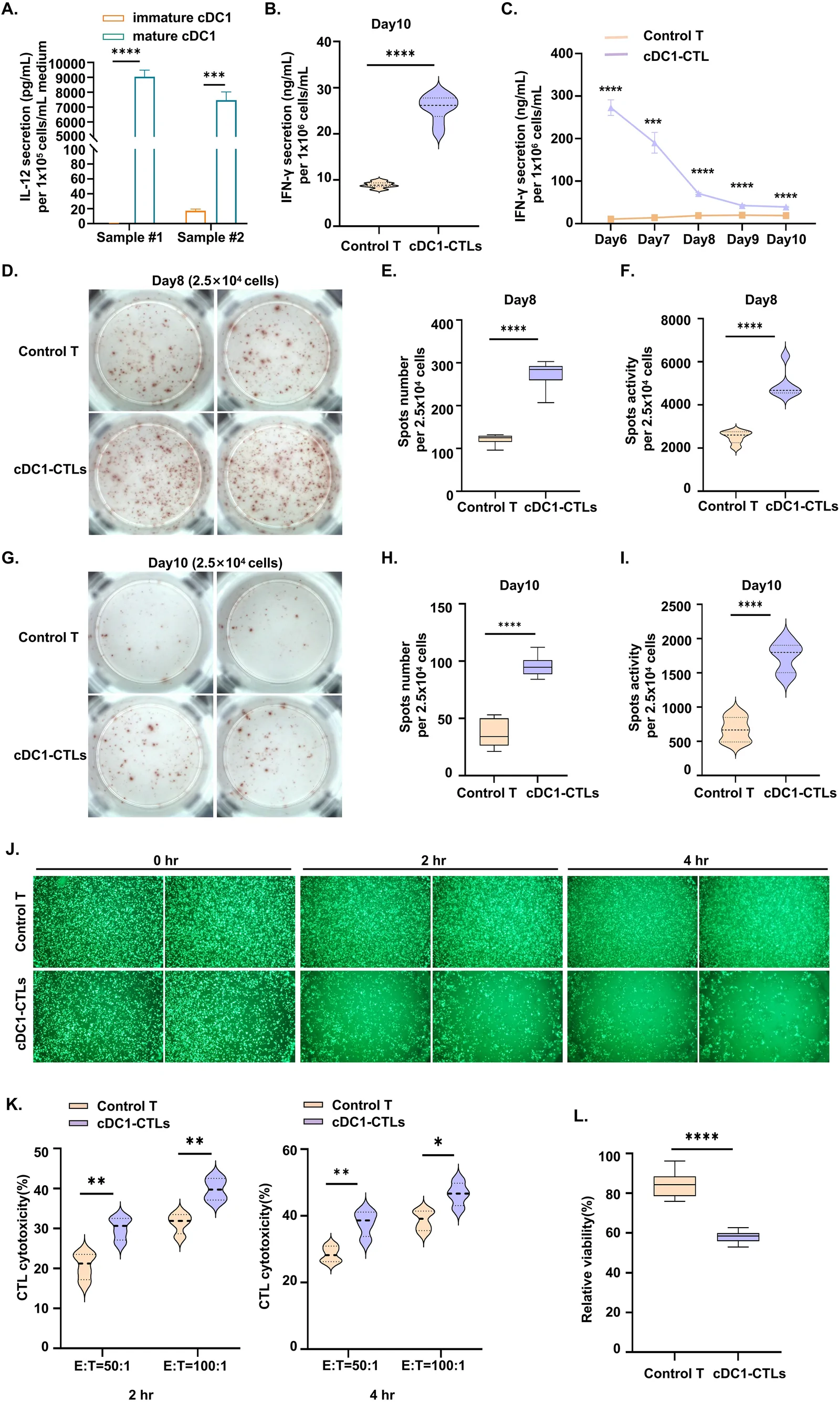
Functional characterization of *in vitro* cultured cDC1 and cDC1 stimulated T cells (cDC1-CTLs). **(A)** ELISA analysis showing that mature cDC1 cells secreted significantly higher levels of IL-12 compared to immature cDC1 cells from two volunteers. ***p < 0.001, ****p < 0.0001. **(B, C)** ELISA analysis demonstrating that cDC1-CTLs secreted significantly more IFN-γ than control T cells on Day 10 for volunteer #1 **(B)**, and from Day 6 to Day 10 for volunteer #2 **(C)**. **(D–F)** IFN-γ ELISpot analysis of control T cells and cDC1-CTLs on Day 8. Representative IFN-γ spot images [**(D)**, n=2], and statistical analysis of spot numbers [**(E)**, n=6] and spot activity [**(F)**, n=6] are shown. ****p < 0.0001. **(G–I)** IFN-γ ELISpot analysis of control T cells and cDC1-CTLs on Day 10. Representative IFN-γ spot images [**(G)**, n=2], and statistical analysis of spot numbers [**(H)**, n=6] and spot activity [**(I)**, n=6] are shown. ****p < 0.0001. **(J, K)** Cytotoxic activity of control T cells and cDC1-CTLs. Labeled target cells (Huh-7) were co-cultured with effector cells (control T or cDC1-CTLs) **(J)**. Effector-to-target cell ratios were 50:1 or 100:1, with incubation times of 2 h or 4 h. Cytotoxicity was calculated for different incubation times [**(K)**, n=4]. **p < 0.01, *p < 0.05. **(L)** CCK8 assay analyzing the viability of Huh-7 cells co-cultured with control T cells or cDC1-CTLs. ****p < 0.0001. Data are presented as mean ± SD; n indicates biological replicates (independent cell preparations from two donors), with donor-level data shown individually. Two-group comparisons were performed using two-tailed unpaired Student’s t-tests.

Next, we assessed IFN-γ production by cDC1-induced CTLs. Compared to unstimulated controls, cDC1-CTLs secreted over threefold higher IFN-γ during a 10-day culture (**Figure 4B**), with peaks of sevenfold and fivefold increases on days 6 and 7, respectively (**Figure 4C**). ELISpot assays confirmed these findings, showing increased IFN-γ spot numbers and intensity at days 8 and 10 (**Figures 4D–I**). Functional assays using Huh7 hepatocellular carcinoma target cells demonstrated that GPC3 peptide–stimulated cDC1-CTLs exhibited significantly greater tumor-cell lysis than control T cells (**Figures 4J, K**). CCK-8 assays showed reduced Huh7 viability to ~58% in cDC1-CTL co-cultures versus ~85% in controls (**Figure 4L**).

These findings strongly suggest that cDC1s robustly activate IFN-γ–secreting, tumoricidal CTLs, underscoring their potential as an effective cellular immunotherapy platform.

### *In vitro*–generated cDC1 cells induce superior activation and cytotoxic function of CTLs compared with conventional moDCs

To compare the functional performance of cDC1s and moDCs, both cell types were co-cultured with monocyte-depleted T cells from two donors (#1 and #2) (**Figure 5A**). Following eight days of culture, the resulting CTLs were assessed both phenotypically and functionally. Flow cytometry revealed that both moDC- and cDC1-stimulated CTLs contained elevated CD8^+^ T and CD16^+^/CD56^+^ NK/NKT populations compared with unstimulated controls. However, cDC1-CTLs exhibited higher enrichment of CD8^+^ T and NK cells, alongside a reduction in NKT frequency (**Supplementary Figure 5**). IFN-γ ELISpot analysis demonstrated significantly higher IFN-γ secretion in cDC1-CTLs than moDC-CTLs, with increased spot counts and cytokine intensity (**Figures 5B–D**). Cytotoxicity assays showed that cDC1-CTLs displayed the strongest tumor-killing activity among all tested groups (**Figures 5E–H**).

**Figure 5:**
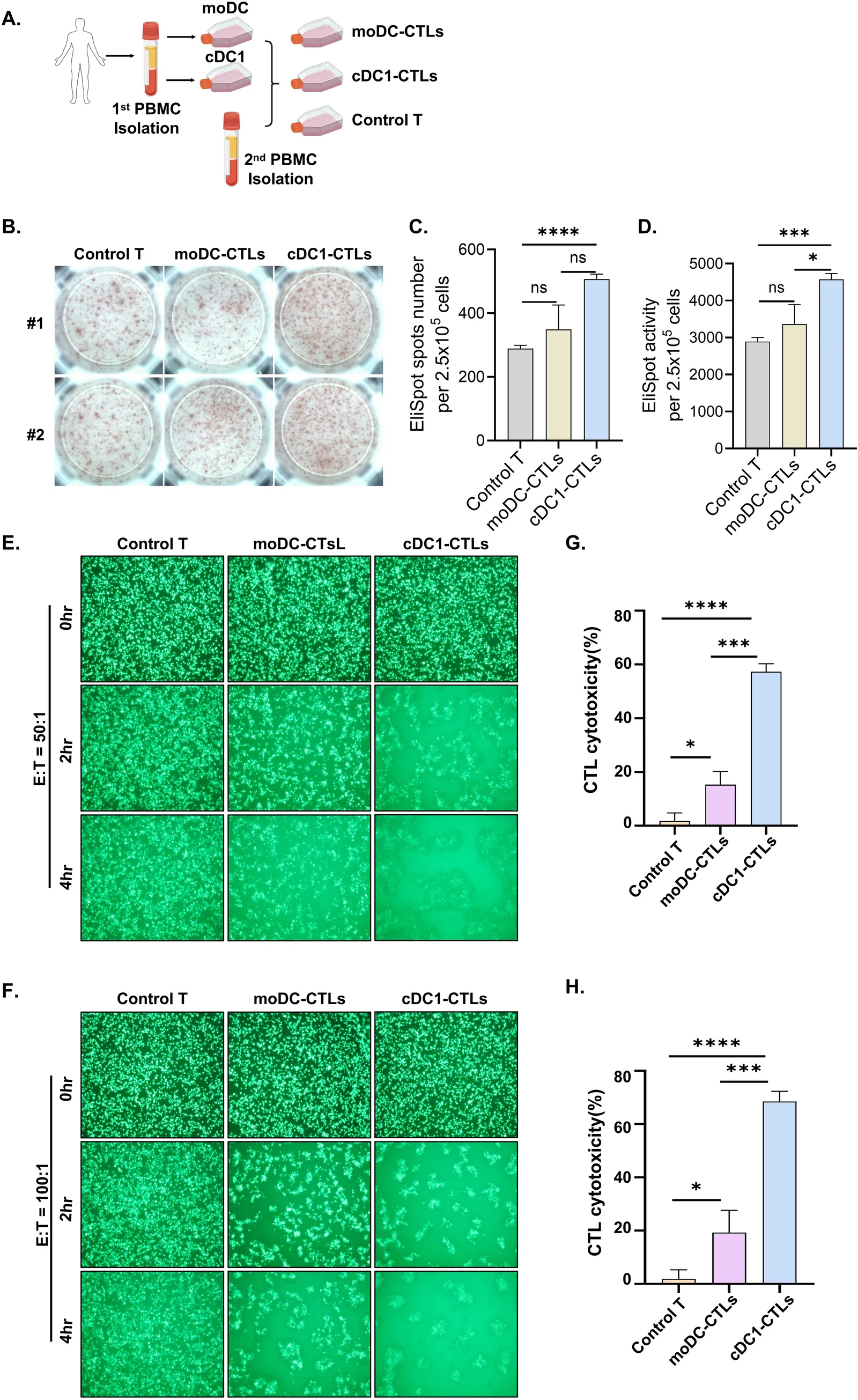
Comparison of cytotoxicity of CTLs obtained by co-culture with cDC1 and moDC, respectively. **(A)** Schematic illustration of control T, moDC-CTLs and cDC1-CTLs production. **(B–D)** IFN-γ ELISpot analysis of control T cells, moDC-CTLs and cDC1-CTLs of Donor #1 and #2 on Day 8. Representative IFN-γ spot images **(B)**, and statistical analysis of spot numbers [**(C)**, n=3] and spot activity [**(D)**, n=3] are shown. ****p < 0.0001, ***p < 0.001, *p < 0.05. **(E–H)** Cytotoxic activity of control T cells, moDC-CTLs and cDC1-CTLs. Labeled target cells (Huh-7) were co-cultured with effector cells (control T, moDC-CTLs and cDC1-CTLs). Effector-to-target cell ratios were 50:1 **(E)** or 100:1 **(F)**, with incubation times of 2 h or 4 h. Cytotoxicity was calculated for the incubation 2 h [**(G, H)**, n=3]. ****p < 0.0001, ***p < 0.001, *p < 0.05. Data are presented as mean ± SD; n indicates biological replicates from two independent donors. Multi-group comparisons were performed by one-way ANOVA followed by Tukey’s multiple-comparisons test.

Aside from CD8^+^ T cells, CD16^+^/CD56^+^ NK/NKT cells were also detected in stimulated cultures (**Supplementary Figure 5**), implying the overall tumor killing effect stems from joint effector cell responses. The utilized GPC3 peptide TIHDSIQYV is HLA-A*02:01-restricted, and GPC3 is abundantly expressed on HepG2 and Huh-7 cells, confirming antigen-specific tumor recognition. Considering this combinatorial effector background, our data preliminarily show that *in vitro*-derived cDC1s achieve better immune activation than moDCs, facilitating the expansion of CD8^+^ CTLs and NK cells with robust anti-tumor activity.

### Preclinical safety evaluation of *in vitro*–generated cDC1 cells

To characterize the preclinical safety profile of GPC3-loaded cDC1s, we carried out a toxicology assessment in NOG mice. Animals received a single subcutaneous injection of either low dose (1 × 10^6^ cells/kg; cDC1-LD) or high dose (1 × 10^7^ cells/kg; cDC1-HD) cDC1s. Vehicle and antigen-only controls (200 μg/kg) were included simultaneously for comparison. Mice were monitored for 14 days to assess acute and delayed toxicities. Our data show that both treatment groups were well tolerated, with no observable toxicities attributable to sex or dosage.

Detailed toxicological analyses—including body weight (**Figures 6A, B**), food intake (**Figures 6C, D**), hematology (**Figures 6E, F**), plasma biochemistry (**Figures 6G, H**), and histopathology (**Supplementary Table 1**) — showed no treatment-related abnormalities. Trace levels of cDC1s were detected in the biceps brachii, heart, brain, blood, and lungs of cDC1-HD animals (**Figure 6I**), without signs of tissue accumulation or proliferation. Cytokine profiling revealed a transient IL-6 elevation—~eightfold on Day 1 and ~threefold on Day 15—while other cytokines (IL-1α, IL-2, IL-4, IL-10, IL-15, IL-18, TNF-α, IFN-γ) remained unchanged or undetectable (**Figure 6J**).

**Figure 6:**
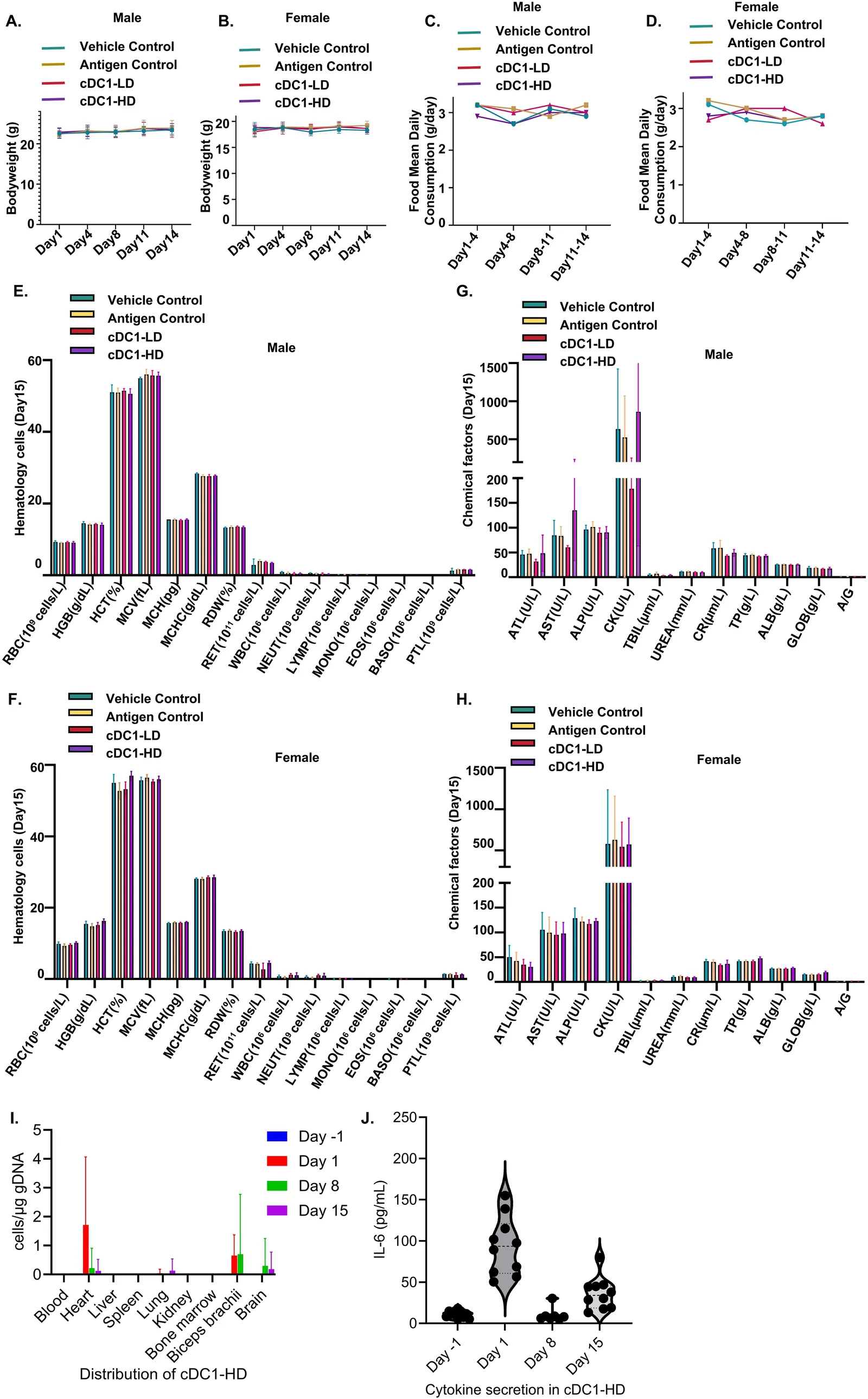
Preclinical safety assessment of *in vitro*-manufactured cDC1 cells. **(A, B)** Body weight profiles of mice treated with *in vitro*-manufactured cDC1 cells. Male **(A)** and female **(B)** mice were evaluated (n=5 per group). **(C, D)** Mean daily food consumption (g/day) in mice treated with *in vitro*-manufactured cDC1 cells. Male **(C)** and Female **(D)** mice were analyzed (n=5 per group). **(E, F)** Hematological parameters (Day 15) of mice treated with *in vitro*-manufactured cDC1 cells. Male **(E)** and Female **(F)** mice were examined (n=5 per group). **(G, H)** Serum chemical factors (Day 15) in mice treated with *in vitro*-manufactured cDC1 cells. Male **(G)** and Female **(H)** mice were assessed (n=5 per group). **(I)** Biodistribution of *in vitro*-manufactured cDC1 cells on Day 1, Day 8, and Day 15. Potential cDC1 cell-derived DNA was detected in blood, heart, liver, spleen, lung, kidney, bone marrow, biceps brachii, and brain tissues. **(J)** Alterations in cytokine (IL-6) levels during cDC1 cell treatment in mice (n=10 per group). Vehicle Control: subcutaneous injections with PBS; Antigen Control: subcutaneous injections with GPC3 antigen (20 μg/ml); cDC1-LD: subcutaneous injections with low-dose cDC1 cells (GPC3 antigen-loaded cDC1, 1×10^6^ cells/kg); cDC1-HD: subcutaneous injections with high-dose cDC1 cells (GPC3 antigen-loaded cDC1, 1×10^7^ cells/kg). Data are presented as mean ± SD (n = 5 mice per sex per group); time-course data were analyzed by two-way ANOVA followed by Šídák’s multiple-comparisons test.

Overall, these findings indicate a favorable short-term safety profile of *in vitro*–generated cDC1 cells in NOG mice. Given the 14-day observation period and the immunodeficient nature of this model, these results should be regarded as preliminary; they support further preclinical development and inform the design of future GLP toxicology and first-in-human studies.

## Discussion

DC-based vaccination shows promise for cancer immunotherapy but faces clinical translation challenges due to rare functional DC subsets in peripheral blood and lack of scalable manufacturing (19). Traditional approaches rely on monocyte-derived DCs (moDCs) which are easy to generate but lack full functionality, while cDC1 generation requires complex conditions or CD34+ progenitors (21) that are less accessible (10). This study presents a streamlined two-step protocol for efficiently generating XCR1^+^CD141^+^ cDC1s from human PBMCs. Transcriptomic profiling confirmed these cells faithfully recapitulate *in vivo* cDC1 molecular signatures, with mature cDC1s showing immune pathway activation and high cytokine secretion including IL-12 critical for Th1 polarization and CTL activation (22). We observed a favorable tendency for antigen-specific CTL priming capacity in these cells; they triggered higher IFN-γ release and stronger tumor cell lytic activity when compared with control T cells and moDC-derived CTLs. This represents an improvement in manufacturing scalability and therapeutic potential over more complex existing methods, although head-to-head comparisons at clinical scale remain to be performed. Preliminary safety evaluation in NOG mice showed no treatment-related toxicity; these findings nevertheless require confirmation in formal GLP toxicology studies.

Despite the promising findings, this study has several limitations. First, most functional experiments were performed with cells from only two donors; although the results were consistent between the two donors, studies with larger donor cohorts will be required to fully establish reproducibility and inter-individual robustness. Second, the CTL cultures generated by cDC1 stimulation contained CD16^+^/CD56^+^ NK/NKT cells in addition to CD8^+^ T cells; therefore, a contribution of NK cells to the observed tumor-cell killing cannot be excluded, and effector-depletion or HLA-blocking experiments will be needed to formally establish GPC3-specific, CD8^+^ T-cell–mediated cytotoxicity. Third, although the *in vitro* priming of CTLs and their tumor-killing activity are encouraging, the efficacy of these cDC1s as a vaccine platform has not yet been assessed in an immunocompetent, tumor-bearing model. This is largely due to the technical challenges of developing humanized systems suitable for both cDC1 generation and *in vivo* efficacy testing, and it limits direct extrapolation of our results to clinical settings. Finally, the safety evaluation was conducted in NOG mice, which lack full adaptive immunity and were monitored for only 14 days; thus, the safety profile observed primarily reflects direct toxicity and acute inflammatory responses rather than the full spectrum of potential immune-mediated or long-term events, and the limitations of this model should be considered when interpreting the translational relevance of these findings.

Overall, this study demonstrates an effective and reproducible strategy for generating functional XCR1^+^CD141^+^ cDC1s from human PBMCs, addressing a long-standing bottleneck in the field and strengthening the foundation for next-generation cDC1-based immunotherapies. These findings support the hypothesis that the enhanced antigen-presentation properties and cytokine profile of these cDC1s may translate into improved antitumor immunity *in vivo* compared with traditional moDC-based vaccines.

Looking ahead, several areas merit further exploration. Human studies will be essential to evaluate safety, immunogenicity, and clinical activity, as well as to refine dosing, delivery, and scheduling parameters. Combination approaches—such as pairing cDC1s with checkpoint inhibitors or cytokine modulators—may further augment therapeutic efficacy (23). Validation of the protocol under real-world GMP manufacturing conditions, together with applications across multiple cancer types and integration with personalized neoantigen platforms, will be important steps toward realizing the full potential of cDC1-based immunotherapy.

## Conclusions

In summary, this study delivers an efficient and reproducible platform for generating human cDC1s, positioning these cells as a promising candidate modality for DC-based cancer immunotherapy. By addressing key limitations in current DC vaccine development—including cell scarcity, donor variability, and manufacturing inconsistency—our work establishes a foundation for the further development of cDC1-based therapies. Going forward, we will advance this platform toward GMP-compliant production, incorporate antigen multiplexing to broaden tumor coverage, and integrate synergistic combinations such as checkpoint inhibitors and oncolytic agents to maximize antitumor efficacy and accelerate clinical translation.

## Data Availability

The datasets presented in this study can be found in online repositories. The names of the repository/repositories and accession number(s) can be found in the article/**Supplementary Material**.
