# Supplementary material for: Efficient expansion of XCR1+CD141+ cDC1 cells from human PBMCs: a promising strategy for DC-based vaccine
Source: Front Immunol. 2026 Oct 19;17:1912573. doi: 10.3389/fimmu.2026.1912573 (PMC13724651; doi:10.3389/fimmu.2026.1912573)
Supplement: Supplementary file 1 [file DataSheet1.pdf]

Figure S1

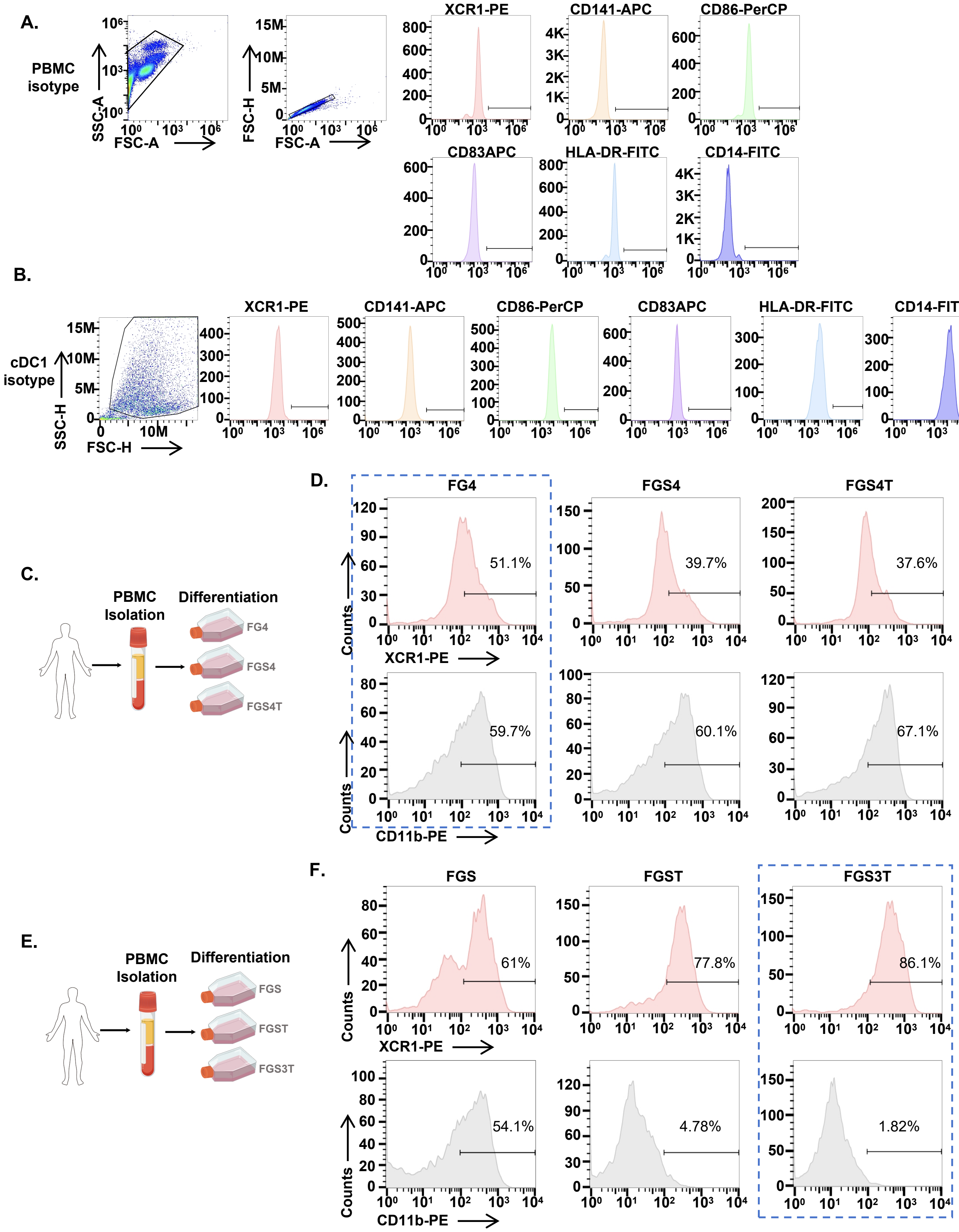

A.
PBMC isotype
SSC-A
FSC-A
FSC-H
XCR1-PE
CD141-APC
CD86-PerCP
CD83APC
HLA-DR-FITC
CD14-FITC
B.
cDC1 isotype
SSC-H
FSC-H
C.
PBMC Isolation
Differentiation
FG4
FGS4
FGS4T
D.
Counts
CD11b-PE
51.1%
39.7%
37.6%
59.7%
60.1%
67.1%
E.
FGS
FGST
FGS3T
F.
61%
77.8%
86.1%
54.1%
4.78%
1.82%

Figure S2

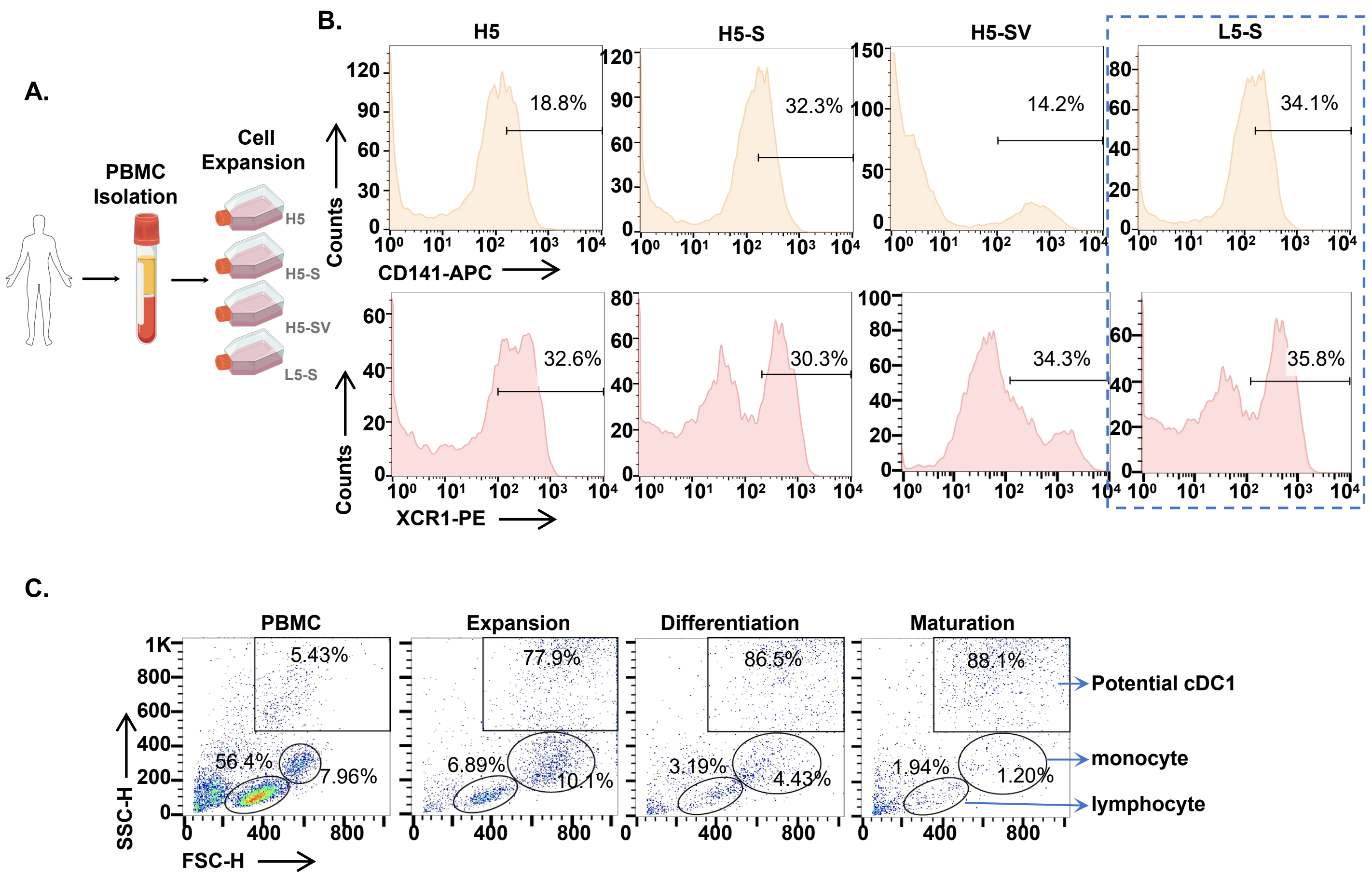
A.
PBMC Isolation
Cell Expansion
H5
H5-S
H5-SV
L5-S
B.
H5
H5-S
H5-SV
L5-S
18.8%
32.3%
14.2%
34.1%
Counts
CD141-APC
32.6%
30.3%
34.3%
35.8%
Counts
XCR1-PE
C.
PBMC
Expansion
Differentiation
Maturation
5.43%
77.9%
86.5%
88.1%
56.4%
7.96%
6.89%
10.1%
3.19%
4.43%
1.94%
1.20%
Potential cDC1
monocyte
lymphocyte
SSC-H
FSC-H

Figure S3

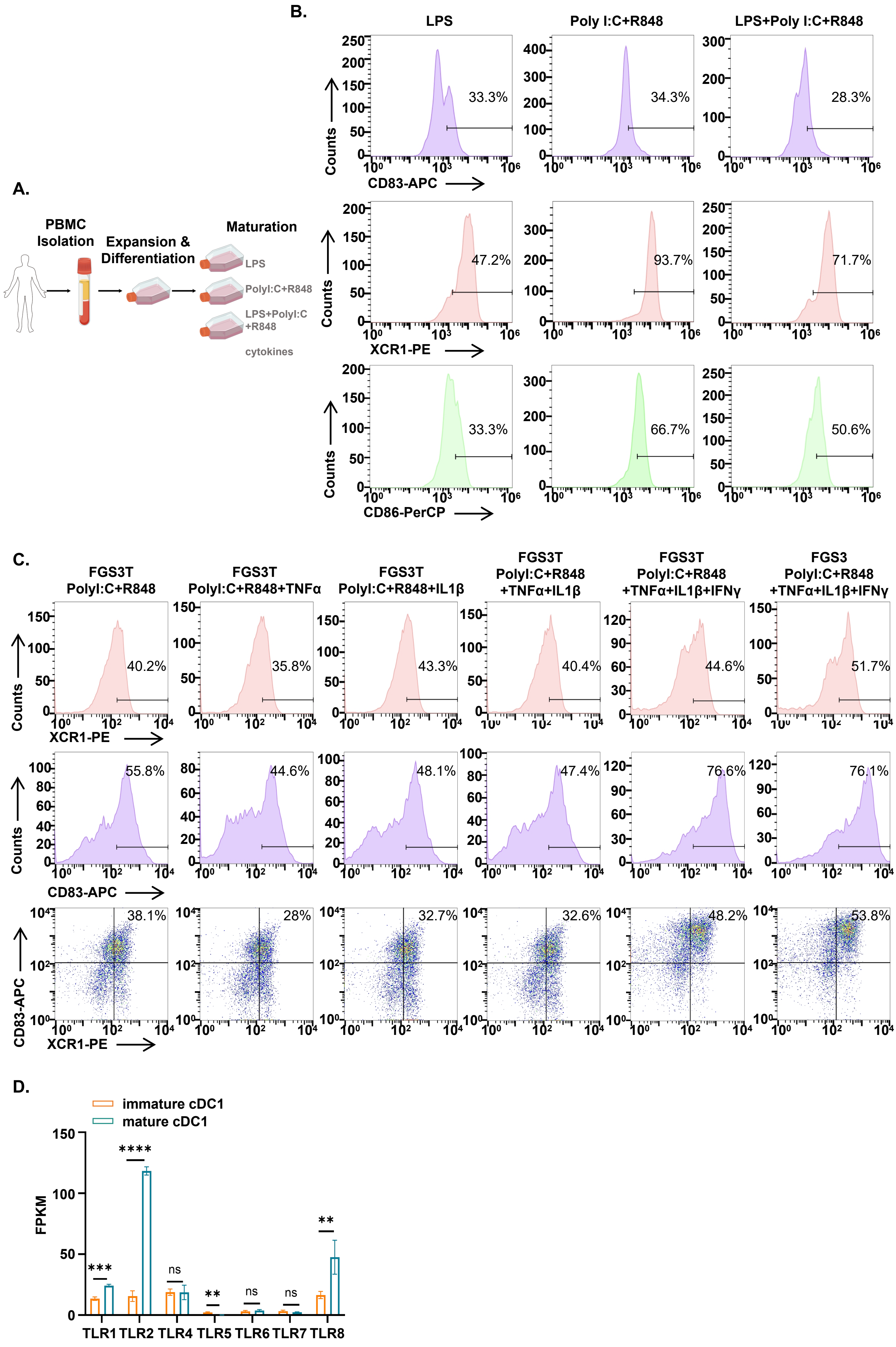

A.
PBMC Isolation
Expansion & Differentiation
Maturation
LPS
PolyI:C+R848
LPS+PolyI:C +R848
cytokines
B.
LPS
Poly I:C+R848
LPS+Poly I:C+R848
Counts
CD83-APC
33.3%
34.3%
28.3%
XCR1-PE
47.2%
93.7%
71.7%
CD86-PerCP
33.3%
66.7%
50.6%
C.
FGS3T PolyI:C+R848
FGS3T PolyI:C+R848+TNFα
FGS3T PolyI:C+R848+IL1β
FGS3T PolyI:C+R848 +TNFα+IL1β
FGS3T PolyI:C+R848 +TNFα+IL1β+IFNγ
FGS3 PolyI:C+R848 +TNFα+IL1β+IFNγ
Counts
XCR1-PE
40.2%
35.8%
43.3%
40.4%
44.6%
51.7%
CD83-APC
55.8%
44.6%
48.1%
47.4%
76.6%
76.1%
38.1%
28%
32.7%
32.6%
48.2%
53.8%
D.
immature cDC1
mature cDC1
FPKM
***
****
ns
**
ns
ns
**
TLR1
TLR2
TLR4
TLR5
TLR6
TLR7
TLR8

Figure S4

A.

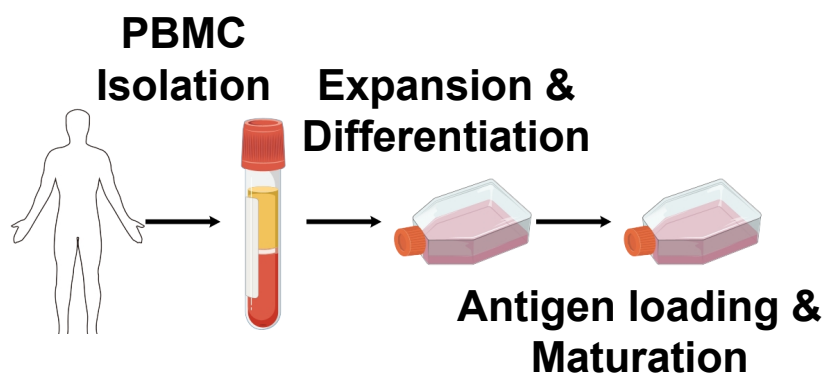
PBMC
Isolation
Expansion &
Differentiation
Antigen loading &
Maturation

B.

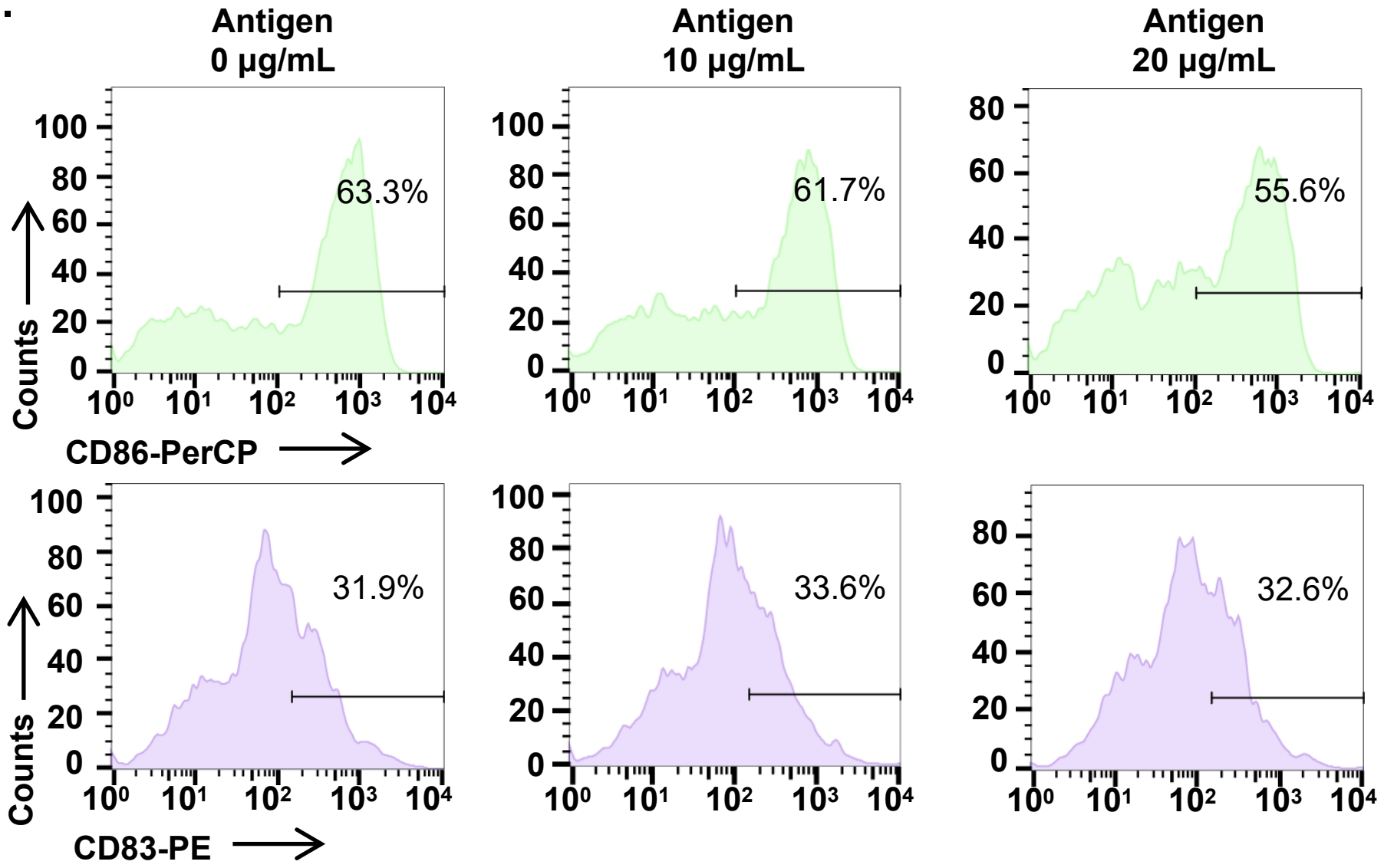
Antigen
0 µg/mL
Antigen
10 µg/mL
Antigen
20 µg/mL
63.3%
61.7%
55.6%
31.9%
33.6%
32.6%
Counts
CD86-PerCP
CD83-PE

Figure S5

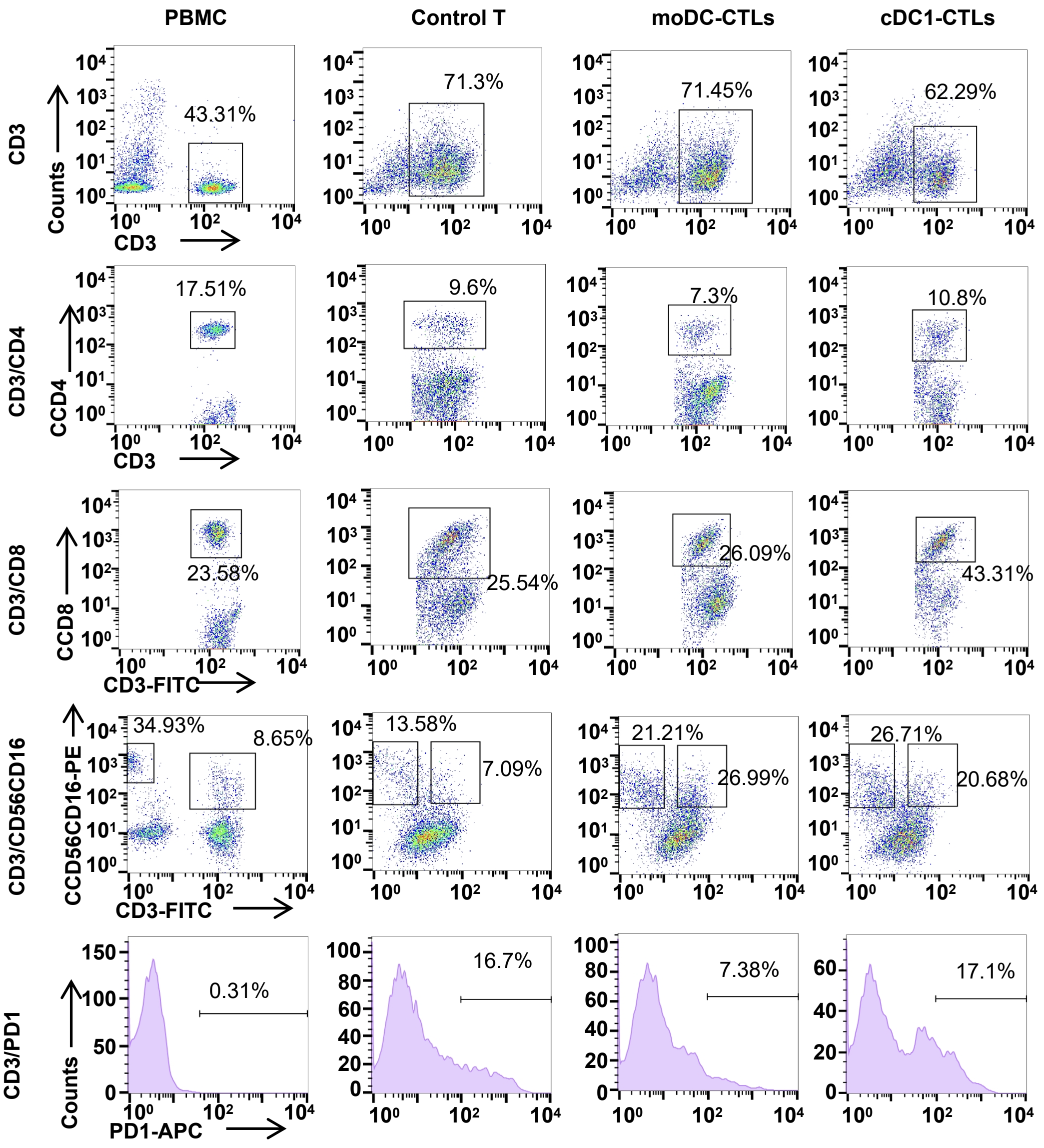
PBMC
Control T
moDC-CTLs
cDC1-CTLs
CD3
Counts
43.31%
71.3%
71.45%
62.29%
CD3/CD4
CCD4
17.51%
9.6%
7.3%
10.8%
CD3/CD8
CCD8
23.58%
25.54%
26.09%
43.31%
CD3-FITC
CD3/CD56CD16
CCD56CD16-PE
34.93%
8.65%
13.58%
7.09%
21.21%
26.99%
26.71%
20.68%
CD3/PD1
PD1-APC
0.31%
16.7%
7.38%
17.1%

Table S1. Summary of pathological results according by anatomical observation.

		Spleen	Heart	Lung	Kidney	Brain	Skeletal Muscle	Femur	Spinal Cord	Pituitary Gland	Thyroid Gland	Salivary Gland	pancreas	Adnimistration stie
Vehicle Control	No Visible Lesions	9/10	10/10	10/10	10/10	10/10	10/10	10/10	10/10	10/10	10/10	10/10	10/10	10/10
	Focal osseous metaplasia	1/10	0/10	0/10	0/10	0/10	0/10	0/10	0/10	0/10	0/10	0/10	0/10	0/10
	Multifocal osseous metaplasia	0/10	0/10	0/10	0/10	0/10	0/10	0/10	0/10	0/10	0/10	0/10	0/10	0/10
Antigen Control	No Visible Lesions	8/10	10/10	10/10	10/10	10/10	10/10	10/10	10/10	10/10	10/10	10/10	10/10	10/10
	Focal osseous metaplasia	2/10	0/10	0/10	0/10	0/10	0/10	0/10	0/10	0/10	0/10	0/10	0/10	0/10
	Multifocal osseous metaplasia	0/10	0/10	0/10	0/10	0/10	0/10	0/10	0/10	0/10	0/10	0/10	0/10	0/10
cDC1-LD	No Visible Lesions	8/10	10/10	10/10	10/10	10/10	10/10	10/10	10/10	10/10	10/10	10/10	10/10	10/10
	Focal osseous metaplasia	2/10	0/10	0/10	0/10	0/10	0/10	0/10	0/10	0/10	0/10	0/10	0/10	0/10
	Multifocal osseous metaplasia	0/10	0/10	0/10	0/10	0/10	0/10	0/10	0/10	0/10	0/10	0/10	0/10	0/10
cDC1-HD	No Visible Lesions	8/10	10/10	10/10	10/10	10/10	10/10	10/10	10/10	10/10	10/10	10/10	10/10	10/10
	Focal osseous metaplasia	0/10	0/10	0/10	0/10	0/10	0/10	0/10	0/10	0/10	0/10	0/10	0/10	0/10
	Multifocal osseous metaplasia	2/10	0/10	0/10	0/10	0/10	0/10	0/10	0/10	0/10	0/10	0/10	0/10	0/10
